# Multicenter Study of Human Papillomavirus and the Human Papillomavirus Vaccine: Knowledge and Attitudes among People of African Descent

**DOI:** 10.1155/2013/428582

**Published:** 2013-07-16

**Authors:** Elizabeth Blackman, Natalie Thurman, Darron Halliday, Raleigh Butler, Dorita Francis, Madeline Joseph, Jahzreel Thompson, Aletha Akers, Cecile Andraos-Selim, Cornelius Bondzi, Emanuela Taioli, Kourtney L. Hagan, Erin A. Jones, Jade Jones, Cierra M. Moss, Ar'Lena C. Smith, Kimlin Tam Ashing, Camille C. Ragin

**Affiliations:** ^1^Cancer Prevention and Control Program, Fox Chase Cancer Center, 333 Cottman Avenue, Philadelphia, PA 19111, USA; ^2^African Caribbean Cancer Consortium, USA; ^3^Department of Epidemiology, University of Pittsburgh, Graduate School of Public Health and the University of Pittsburgh Cancer Institute, Pittsburgh, PA, USA; ^4^Department of Obstetrics & Gynecology, Princess Margaret Hospital, Nassau, Bahamas; ^5^Department of Family Medicine, Princess Margaret Hospital, Nassau, Bahamas; ^6^Division of Gynecologic Specialties, Department of Obstetrics, Gynecology, and Reproductive Sciences, University of Pittsburgh, Pittsburgh, PA, USA; ^7^Department of Biological Sciences, Hampton University, Hampton, VA, USA; ^8^North Shore Long Island Jewish Health System, The Feinstein Institute for Medical Research, 350 Community Drive, Manhasset, NY, USA; ^9^Division of Cancer Prevention and Population Science, University of Pittsburgh Cancer Institute, Pittsburgh, PA, USA; ^10^Center of Community Alliance for Research and Education, Department of Population Science, City of Hope Medical Center, Duarte, CA, USA

## Abstract

*Objective*. To compare knowledge and attitudes of human papillomavirus (HPV) and the vaccine between different cultures of African descent. *Methods*. A cross-sectional survey of 555 African-Americans and Afro-Caribbeans residing in the US and the Bahamas (BHM) was conducted. *Results*. General knowledge about HPV and the HPV vaccine differed between the two countries significantly. Bahamian respondents were less likely to have higher numbers of correct knowledge answers when compared to Americans (Adjusted Odds Ratio [Adj. OR] 0.47, 95% Confidence Interval [CI] 0.30–0.75). Older age, regardless of location, was also associated with answering fewer questions correctly (Adj. OR 0.61, 95% CI 0.40–0.92). Attitudes related to HPV vaccination were similar between the US and BHM, but nearly 80% of BHM respondents felt that children should not be able to receive the vaccine without parental consent compared to 57% of American respondents. *Conclusions*. Grave lack of knowledge, safety and cost concerns, and influence of parental restrictions may negatively impact vaccine uptake among African-American and Afro-Caribbean persons. Interventions to increase the vaccine uptake in the Caribbean must include medical provider and parental involvement. Effective strategies for education and increasing vaccine uptake in BHM are crucial for decreasing cervical cancer burden in the Caribbean.

## 1. Introduction

Cervical cancer (CC) is listed among the top five cancers that affect women globally. According to the International Agency for Research on Cancer (IARC), in 2008 there were 530,000 new cases of cervical cancer worldwide, with 85% of the disease burden occurring in developing countries. Caribbean women are at an increased risk of dying from CC, which is the second most frequent cancer among women and also ranks as the third most frequently diagnosed cancer in both sexes [1]. In the United States (US), CC has decreased in incidence and mortality since the mid 19th century, primarily because of screening [2]. Even with the introduction and widespread use of the Pap test, CC still ranks among the top ten cancers diagnosed in the US among minority populations, which includes Blacks, American Indians, and Hispanics [3].

Persistent high-risk (HR) human papillomavirus (HPV) infection, specifically HPV types 16 and 18, has been linked to the development of CC, anogenital cancers, and oropharyngeal cancers [4, 5]. In 2006, the FDA approved the HPV vaccine *Gardasil *(against HR HPV types 16 and 18 as well as low-risk types 6 and 11) for all females aged 9 through 26. In 2009, the FDA also approved the use of this vaccine in males aged 9 through 26 [6]. *Cervarix*, which aims to protect against HR HPV types 16 and 18 only, was approved by the FDA in the Fall of 2009 for females aged 10 through 25 [7]. The Centers for Disease Control and Prevention (CDC) Advisory Committee on Immunization Practices (ACIP) recommended routine HPV vaccination for adolescent boys and girls as young as 9 years and men and women as old as 26 [8]. However, even with the availability of these vaccines, there is still a disparity in their utilization between races. Minorities are not receiving and/or completing the vaccine process at the same rate that their White counterparts are in the US. A study conducted among 363 African-American college females showed that only about 25% of young women report uptake of the HPV vaccine. Vaccine uptake was associated with significantly higher levels of HPV knowledge, lower perceived barriers to vaccination, and younger age [9]. With respect to vaccine completion, Niccolai et al. reported that completing the vaccination series was associated with being White and having an annual household income >$75,000 [10].

 Reasons as to why disparity exists for HPV vaccine uptake and completion remain unclear in the literature. Some studies attribute racial disparity to income level differences, while others believe it is due to lack of knowledge [10–12]. The poor response and completion rate of HPV vaccination amongst minorities still need to be fully assessed. In our earlier study of HPV knowledge amongst the general population, we reported that the general population was aware of HPV as well as the HPV vaccine; however, the benefits of the vaccine to the study population were not evident. When looking at knowledge according to race, people of African descent were less informed than Whites (Black 89% versus White 97%, *P* > 0.1) [13].

 Cultural beliefs or perceptions and lifestyle are distinct between persons born in the US and those born in other countries [14–16]. While there is a similar concern for high CC incidence among Black women in the Caribbean and the US, interventions that promote HPV vaccine uptake and completion may need to be tailored differently for each group. Prior to the development of needed interventions, information on knowledge and attitudes toward HPV and its vaccine is needed. Yet, no previous publication has assessed HPV knowledge and/or attitude towards HPV vaccination in the Caribbean. Therefore we have expanded our previously published study to include an example of a Caribbean nation (the Bahamas) where CC incidence (17.6 per 100,000) is similar to the incidence of CC for the Caribbean region (20.8 per 100,000). We sought to determine knowledge and attitudes of HPV and the vaccine in the Bahamas and determine whether differences exist between different cultures of African descent.

## 2. Methods

### 2.1. Study Population

The original study population consisted of 202 volunteer participants from the University of Pittsburgh and Hampton University [13]. The recruitment was expanded and involved two additional locations: the New York City (NYC) metropolitan area and The Bahamas (BHM), West Indies. Therefore, this study includes a diverse study population recruited from the general population between 2008 and 2010 from three urban cities in the North Eastern US differing in size and demographics, (small (Hampton, VA, population 136,401) medium (Pittsburgh, PA, population 307,484) and large (NYC, NY, population 8.245 million). Both Hampton, VA and Pittsburgh, PA, comprise more of an African-American demographic compared to NYC where a higher proportion of the population consists of immigrant families, including the Caribbean. We believe that this mix is diverse and may be more representative of the US population. Institutional Review Board approval was obtained from the University of Pittsburgh, SUNY Downstate Medical Center, and Princess Margaret Hospital in the Bahamas. For all study locations, study participants were recruited face-to-face or using posted flyers in various settings including hospitals, private and other clinics, and community locations (e.g., parks, restaurants, barber shops and laundromats, etc.). The original English questionnaire was formally translated into Haitian Creole by the Haitian Embassy in the Bahamas in order to accommodate Creole-speaking participants. Those who were not able to read or write well had assistance in completing the questionnaire. All surveys were completed without including identifiable information for the study participants. A total of 793 surveys were administered and returned; of these, 231 were excluded from the present analysis because they were completed by people who identified themselves as some race other than Black. The study population was then divided into two groups based on place of current residence—US and West Indies.

### 2.2. Questionnaire

The questionnaire used in this study consisted of demographic questions pertaining to age, race, sex, place of birth, religion, household income, level of education, health insurance status, parental status, and number of children. The study instrument included measures of HPV knowledge and the safety, efficacy, and impact of the HPV vaccine. A content assessment of the survey was conducted prior to the launch of the study [13].

### 2.3. Statistical Analysis

Descriptive statistics were generated for demographic and knowledge variables according to the place of residence for study participants. Fisher's exact test was used to determine statistical significant differences between proportions; differences in continuous variables were assessed using two-sample *t*-tests. Adjusted prevalence of correct answers to HPV and HPV vaccine knowledge questions was calculated and stratified by geographic location after adjusting for age, level of education, marital status, parental status, health insurance status, and income level. Ordered logistic regression was performed to assess differences in response to knowledge questions pertaining to HPV and HPV vaccination between the two groups, adjusting for demographic variables and the covariates previously mentioned. Statistical analysis was performed using STATA version 10.1 software (Stata LP, College Station, TX, USA). A *P* value of ≤0.05 was considered statistically significant.

## 3. Results

### 3.1. Study Population

A total of 555 Black participants residing in the US and the Bahamas (BHM) completed the survey (41% from the US and 59% from the BHM (Table 1)). Between both populations, 4% of the subjects (*n* = 23) were immigrants and not born in their current country of residence. Twenty-five percent of the participants were male, and 75% were female; there was no difference in gender between the two geographic locations. In both locations, the majority of the participants were between the ages of 18 and 35, with a higher proportion of this age group in the US sample compared to the BHM (80.5% versus 49%, *P* < 0.0001). Eighty-four percent of the US population was single compared to only 42% of the BHM population. Parenthood also demonstrated differences, where more Bahamians tended to be parents (BHM: parent = 65.2% versus US: parent = 23.9%, *P* < 0.0001). The correlation between parental status and marital status also differed between the US and Bahamas. The majority of Bahamian parents were married (45%), while the majority of US parents were single (41%). All US respondents completed high school, while 11.5% of Bahamians did not. US participants were more likely to have attended or completed college (US: some college = 55.3%, college graduate = 27.4% versus BHM: some college = 17.9%, college graduate = 22.0%, *P* < 0.001). Income levels varied modestly between groups up to the US $50,000 mark; 40.7% of US respondents fell into this bracket compared to 11.7% of Bahamian respondents reporting this as their income.

### 3.2. HPV: Virus and Vaccination Knowledge

General knowledge about HPV and the HPV vaccine differed between the two countries significantly (Table 2). When asked if they ever heard of HPV, Bahamian participants were less aware of the virus than participants in the US (US 89.5% versus BHM 61.5%, *P* < 0.001). This difference in knowledge remained consistent for 6 out of the 10 questions related to HPV knowledge, while there was no difference in knowledge for four specific questions. Although the majority of respondents from both locations knew that only women could develop cervical cancer, lower proportions of respondents knew that HPV causes genital warts (28.2% of US and 33.2% of Bahamians) and that genital warts are not caused by the same HPV types that cause cervical cancer (13.8% of Americans and 7.5% of Bahamians).

For the questions related to HPV vaccination (Table 3), the majority of both populations heard of the vaccine, however, the proportion was higher among American respondents (US 66.9% versus BHM 50.6%, *P* = 0.021).

For two of the three questions assessing knowledge about the HPV vaccine (Table 3), Bahamian study participants were more likely to answer questions incorrectly when compared to American Blacks. At the time the questionnaire was administered, the HPV vaccine had not yet been approved for males; when asked who was eligible to receive vaccination, approximately 51% of Americans answered correctly versus only 35% of Bahamian participants (*P* = 0.013). More than half of both populations knew that, in spite of vaccination, annual Pap smears were still needed to screen for cervical cancer according to the recommendation at that time. Even so, a significantly lower proportion of Bahamians answered this question correctly (US 75.3% versus BHM 51.5%, *P* < 0.0001). There was no difference between the two groups when asked what age group was eligible to receive the vaccine; less than half of each group answered with the correct age group, 9–26 (US 47.6% versus BHM 40.4%, *P* = 0.264).

**Figure 1:**
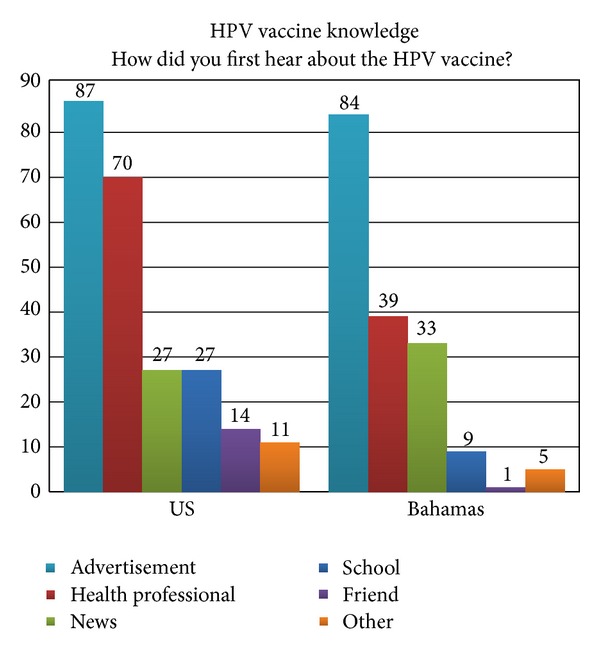
HPV vaccine knowledge: where did you learn about the HPV vaccine? Distribution of those who heard of the HPV vaccine by the source of knowledge, stratified by geographic location.

 For the participants that had previous knowledge of the HPV vaccine, the source of this information was similar between the two groups (Figure 1). For the US, HPV vaccine awareness was first provided through an advertisement followed by a health care professional, the news, and school, although, in the Bahamas, the primary mechanisms for promoting awareness were advertisement followed by a health care professional and the news with minimal contribution from school.

Table 4 shows cumulatively the number of answers that respondents from each geographic location answered correctly. After adjusting for covariates (Table 5), Bahamian respondents were less likely to have higher numbers of correct knowledge answers when compared to Americans (Adjusted Odds Ratio [Adj. OR] 0.47, 95% Confidence Interval [CI] 0.30–0.75). Older age, regardless of location, was also associated with answering fewer questions correctly (Adj. OR 0.61, 95% CI 0.40–0.92). Having health insurance and higher income levels, regardless of location, was associated with answering higher numbers of questions correctly (Adj. OR 1.76, 95% CI 1.07–2.92; Adj. OR 1.21, 95% CI 1.08–1.35, resp.).

### 3.3. HPV Vaccine Attitudes

**Figure 2:**
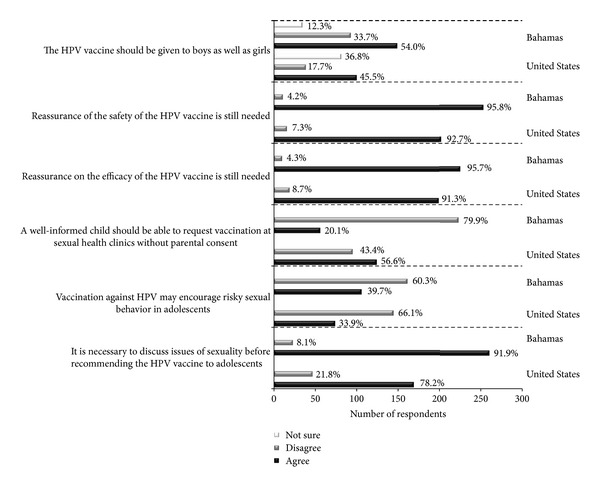
Attitudes towards HPV vaccination. Responses to questions that evaluated attitudes towards HPV vaccination, stratified by geographic location.

When asked whether or not the HPV vaccine should be given to both boys and girls, both US and BHM participants had mixed views; however, the majority of both groups agreed that both genders should receive the vaccine (Figure 2). For both US and Bahamian parents, the majority indicated that they were willing to vaccinate their daughters (US = 21/51, 41% and Bahamas = 96/187, 51%). For the US parents unwilling to vaccinate their daughters, the majority reported that their reason was safety concerns (data not shown). However, it was not possible to make comparisons between the two populations since 66% of the Bahamian parents who were unwilling to vaccinate their daughters did not indicate the reason. For both groups, 90% or more respondents agreed that assurances were still needed for vaccine safety and efficacy. Overall, the majority of participants from both populations did not feel that administration of the vaccine would encourage risky sexual behavior and felt that discussing issues of sexuality before vaccination was necessary. When asked if an informed child should be able to request vaccination at sexual health clinics without parental consent, nearly 80% of Bahamian respondents felt that children should not be able to receive the vaccine without parental consent compared to 57% of American respondents.

## 4. Discussion

A number of international studies have focused on knowledge and attitudes related to HPV and the HPV vaccine. However, a majority of these studies have been conducted in Europe, Asia, and Africa [17–23], with little work done in Latin America [11] and no studies conducted in the Caribbean. This study is the first to report on overall HPV and HPV vaccine knowledge and perception in a sample of Black volunteers containing a large number of subjects from the US and Caribbean, and we have compared our findings by geographic residence. Our previous study showed a lack of knowledge among people of African descent in the US, which remained consistent with our current findings and other published literature [24, 25]. However, the present study indicates that overall knowledge was significantly lower in Bahamian participants when compared to American Blacks, even after adjusting for possible confounders.

 More than half of participants from each location were aware that a vaccine for HPV is available; however Bahamian participants were less aware of this. Studies that focused on populations of African descent, outside of the US, report a similar lack of knowledge in regard to vaccine availability [21, 26], and educational attainment has been shown to be associated with familiarity of HPV and its vaccine [23, 25, 27]. In our study population, US participants had higher levels of education compared to Bahamian participants, which could explain the lesser familiarity with HPV and its vaccine in the Bahamas compared to the US. For persons who were aware of the HPV vaccine, there were slight differences between the US and Bahamas in the type of resource from which they received this information. While advertisements were the major resource for obtaining information about HPV vaccines for both the US and Bahamian populations, health professionals were also an important resource for the US population but not as much for the Bahamas. Further investigations that help clarify the reasons why health professionals are not currently important resources for educating the Bahamian population about HPV vaccines are warranted and might help provide insight for improving HPV vaccine awareness in this geographic region. 

 Published literature suggests that in most cases, after parents were well informed about the risks and benefits of the vaccine, they were willing to vaccinate their children [11, 19, 28], and we have noted that in our study the majority of parents were willing to vaccinate their daughters. For the US, parents who were unwilling to vaccinate their daughters identified their reasons as safety concerns. However, in this study we were not able to compare the reasons for unwillingness of parents to vaccinate their children between the two countries, because of low response rates to those questions for the Bahamian parents. It was not clear why the response rate for this question was low; however, it is possible that the participants may not have been comfortable with disclosing their reasons.

 In general, attitudes related to HPV vaccination were similar between the US and BHM but differed when the two populations were asked if a well-informed child should be able to request vaccination at sexual health clinics without parental consent. US participants were more likely to agree with this statement (*P* < 0.001). This difference may suggest that Americans have a higher self-worth in regard to their health, which is not the case when it comes to children. Hughes reports that children considered themselves to be passive participants when determining their course of medical care [29]. In our study population, the majority of parents from BHM were married in contrast to the parents from the US. In the West Indies, the importance of family has been distinguished and deemed quite atypical to that of the western world. Although speculative, the rationale behind the majority of Bahamian respondents disagreeing with children being able to receive the vaccine without parental consent might be explained by differences in overall family structure and child rearing practices, which have been shown to differ when compared to the US [30]. Still, with the spread of American culture globally, family values have begun to change within the Caribbean [30], which would explain why younger respondents from BHM tended to agree with the statement.

 Respondents who agreed with the statement about children being able to decide about their own vaccination tended to be younger people (<35 years), regardless of geographic location (*P* < 0.001).

## 5. Conclusions

Lack of knowledge in regard to HPV and the HPV vaccine is not a problem that is limited to peoples of African descent but extends to ethnicities within developing countries all over the world. Regardless of geographic location, Whites have consistently displayed higher levels of knowledge concerning HPV and the vaccine when compared to other ethnic groups [13, 26]. Effective methods in disseminating necessary information to these populations with lower health education need to be assessed and put into action to minimize future cases of HPV-related CC. Given that the majority of study participants first heard about the HPV vaccine via advertisements or their health care providers, design of an intervention that combines the two may be effective in reaching the public. In cases where access to health care providers is limited, the use of community-based educators may prove to be more effective than gaining information from a physician. Moreover study findings suggest that interventions to increase the uptake of HPV vaccine in the Caribbean must include parental involvement. In summary, the loss of productive lives and quality of life caused by morbidity and early death from mortality due to cervical cancer in the Caribbean and Latin American region is significant. Therefore, the need for strategies to prevent HPV infection is urgent and compelling. Determining the best strategies to educate and increase HPV prevention practices in this population is crucial and needs to be treated with a sense of exigency in order to decrease the burden of CC in this region.

## Figures and Tables

**Table 1:** Description of study population.

	US Black participants (%)	West Indian participants (%)	*P* value
Age (years)	*n* = 226	*n* = 325	<0.0001
18–35	182 (80.5)	160 (49.2)	
36–55	38 (16.8)	139 (42.8)	
56+	6 (2.7)	26 (8)	

Gender	*n* = 226	*n* = 323	0.385
Male	62 (27.4)	78 (24.1)	
Female	164 (72.6)	245 (75.9)	

Education	*n* = 226	*n* = 323	<0.0001
Less than HS	0 (0.0)	37 (11.5)	
High school	23 (10.2)	101 (31.3)	
Some college	125 (55.3)	58 (17.9)	
College	62 (27.4)	71 (22.0)	
Graduate school	16 (7.1)	56 (17.3)	

Marital status	*n* = 227	*n* = 328	<0.0001
Married	23 (10.1)	108 (32.9)	
Single	192 (84.6)	137 (41.8)	
Other	12 (5.3)	83 (25.3)	

Parent	*n* = 213	*n* = 287	<0.0001
Yes	*51 (23.9) *	*187 (65.2) *	
No	*162 (76.1) *	*100 (34.8) *	

Income	*n* = 204	*n* = 264	<0.0001
Less than US$15,000	49 (24.0)	61 (23.1)	
US$15,000–US$25,000	24 (11.8)	77 (29.2)	
US$25,000–US$35,000	17 (8.3)	54 (20.5)	
US$35,000–US$45,000	18 (8.8)	20 (7.6)	
US$45,000–US$55,000	13 (6.4)	21 (7.9)	
Above US$55,000	83 (40.7)	31 (11.7)	

Health insurance status	*n* = 221	*n* = 315	<0.0001
Uninsured	15 (6.8)	167 (53.0)	
Insured	206 (93.2)	148 (47.0)	

**Table 2:** HPV knowledge. Adjusted for age, level of education, marital status, parental status, health insurance status, and income level.

	US % (95% CI)	Bahamas % (95% CI)	*P* value
Have you heard of the human papillomavirus (HPV)? *Answer = yes *	89.5 (82.4–93.9)	61.5 (52.3–69.9)	0.000
HPV is not a sexually transmitted disease. *Answer = false *	51.5 (42.3–60.7)	31.4 (24.6–39.2)	0.002
HPV is a relatively uncommon disease. *Answer = false *	69.2 (60.3–76.9)	40.6 (33.0–48.7)	0.000
HPV causes cervical cancer. *Answer = true *	69.8 (60.1–77.7)	53.9 (45.5–62.0)	0.019
Who can become infected with HPV? *Answer = both men and women *	42.5 (34.0–51.5)	31.5 (24.8–39.0)	0.076
Both men and women can have cervical cancer. *Answer = false *	69.1 (60.2–76.7)	64.9 (56.9–72.1)	0.512
The incidence of HPV in women is highest among women in their 20s and 30s. *Answer = true *	56.3 (46.9–65.3)	29.2 (22.4–36.9)	0.000
Most people with genital HPV infections are symptomatic. *Answer = no *	35.5 (27.3–44.6)	20.7 (15.1–27.6)	0.009
HPV causes genital warts. *Answer = true *	28.2 (21.1–36.6)	33.2 (26.1–41.1)	0.400
Genital warts are caused by the same HPV types that cause cervical cancer. *Answer = false *	13.8 (8.8–20.9)	7.5 (4.5–12.4)	0.066
There is a cure for HPV infection. *Answer = no *	37.2 (28.6–46.6)	22.6 (16.6–30.0)	0.013

**Table 3:** HPV vaccine knowledge. Adjusted for age, level of education, marital status, parental status, health insurance status, and income level.

	US % (95% CI)	Bahamas % (95% CI)	*P* value
Have you heard about the HPV vaccine? *Answer = yes *	66.9 (57.3–75.3)	50.6 (42.0–59.1)	0.021
Who is eligible for the HPV vaccine? *Answer = women* ^a^	51.0 (42.2–59.7)	35.2 (28.4–42.7)	0.013
For which age group is the HPV vaccine recommended? *Answer = 9 through 26 *	47.6 (38.9–56.6)	40.4 (33.0–48.2)	0.264
Once vaccinated, women no longer have to be screened (annual Pap smears) for cervical cancer. *Answer = false *	75.3 (66.9–82.1)	51.5 (43.6–59.4)	0.000

^a^At the time the survey was administered, no HPV vaccine was FDA approved to be administered to males.

**Table 4:** Correct answers to overall HPV and vaccination knowledge questions.

Number of correct answers	US participants *n* = 227	Caribbean participants *n* = 328	
0-1	14 (6.2)	152 (46.3)	<0.0001
2–7	64 (28.2)	85 (25.9)	
8–11	92 (40.5)	52 (15.9)	
12 or more	57 (25.1)	39 (11.9)	

**Table 5:** Ordered logistic regression for number of correct answers.

	Odds Ratio	95% Confidence Interval
Age (18–35 versus >35 years)	0.61	0.40–0.92
Level of education (<HS versus >HS)	2.12	1.72–2.61
Marital status (married versus single and other)	1.01	0.74–1.39
Parental status (nonparent versus parent)	0.50	0.31–0.82
Level of income (<US$15,000 versus >US$15,000)	1.21	1.08–1.35
Insurance status (no insurance versus insurance)	1.76	1.07–2.92
Place of residence (US versus BHM)	0.47	0.30–0.75

## Abbreviations

ACIP: Advisory Committee on Immunization Practices
Adj. OR: Adjusted Odds Ratio
BHM: Bahamas
CC: Cervical cancer
CDC: Centers for Disease Control and Prevention
CI: Confidence interval
HPV: Human papillomavirus
HR: High risk
IARC: International Agency for Research on Cancer.
